# Metabolic acidemia due to saline absorption during transurethral and transcervical surgery: a report of 2 cases

**DOI:** 10.1186/s12871-024-02437-5

**Published:** 2024-02-10

**Authors:** Mizuyuki Nakamura, Kohei Ikeda, Shoichi Uezono

**Affiliations:** Department of Anesthesiology, The Jikei University School of Medicine, Nishi-Shimbashi 3-25-8, Minato-ku, Tokyo, 105-8461 Japan

**Keywords:** Hyperchloremic metabolic acidosis, Metabolic acidemia, Transurethral resection, Transcervical resection, Saline irrigation, Irrigation fluid absorption

## Abstract

**Background:**

The development of endoscopic systems that include bipolar electrocautery has enabled the use of normal saline irrigation in transurethral or transcervical endoscopic surgery. However, excessive saline absorption can cause hyperchloremic metabolic acidosis.

**Case presentation:**

Patient 1: A 76-year-old man was scheduled for transurethral resection of the prostate with saline irrigation. Approximately 140 min after the surgery, abdominal distension and cervical edema were observed. Abdominal ultrasound examination indicated a subhepatic hypoechoic lesion, which suggested extravasation of saline. Arterial blood gas analysis revealed hyperchloremic metabolic acidosis. The patient was extubated 2 h after the operation with no subsequent airway problems, and the electrolyte imbalance was gradually corrected. Patient 2: A 43-year-old woman was scheduled for transcervical resection of a uterine fibroid with saline irrigation. When the drape was removed after the operation was finished, notable upper extremity edema was observed. Arterial blood gas analysis revealed hyperchloremic metabolic acidosis. The patient’s acidemia, electrolyte imbalance, and neck edema gradually resolved, and the patient was extubated 16 h after the operation without subsequent airway problems.

**Conclusions:**

Anesthesiologists should be aware of acidemia, cardiopulmonary complications, and airway obstruction caused by excessive saline absorption after saline irrigation in endoscopic surgery.

## Background

Non-electrolyte hypo-osmolar irrigation fluid is commonly used in transurethral or transcervical endoscopic surgeries, and a large amount of irrigation fluid absorption (IFA) through vessels can result in transurethral resection (TUR) syndrome, characterized by dilutive hyponatremia, circulating blood volume overload, and disturbance of consciousness [1]. The recent widespread adoption of endoscopic systems that allow bipolar electrocautery and normal saline irrigation in transurethral and transcervical endoscopic surgeries has eliminated complications such as TUR syndrome [2]. However, the potential for additional electrolyte and acid‒base imbalances should be considered when irrigating with normal saline. We hereby report 2 cases of metabolic acidemia caused by the absorption of normal saline during transurethral and transcervical endoscopic surgeries. 

## Case presentation

### Patient 1

**Fig. 1:**
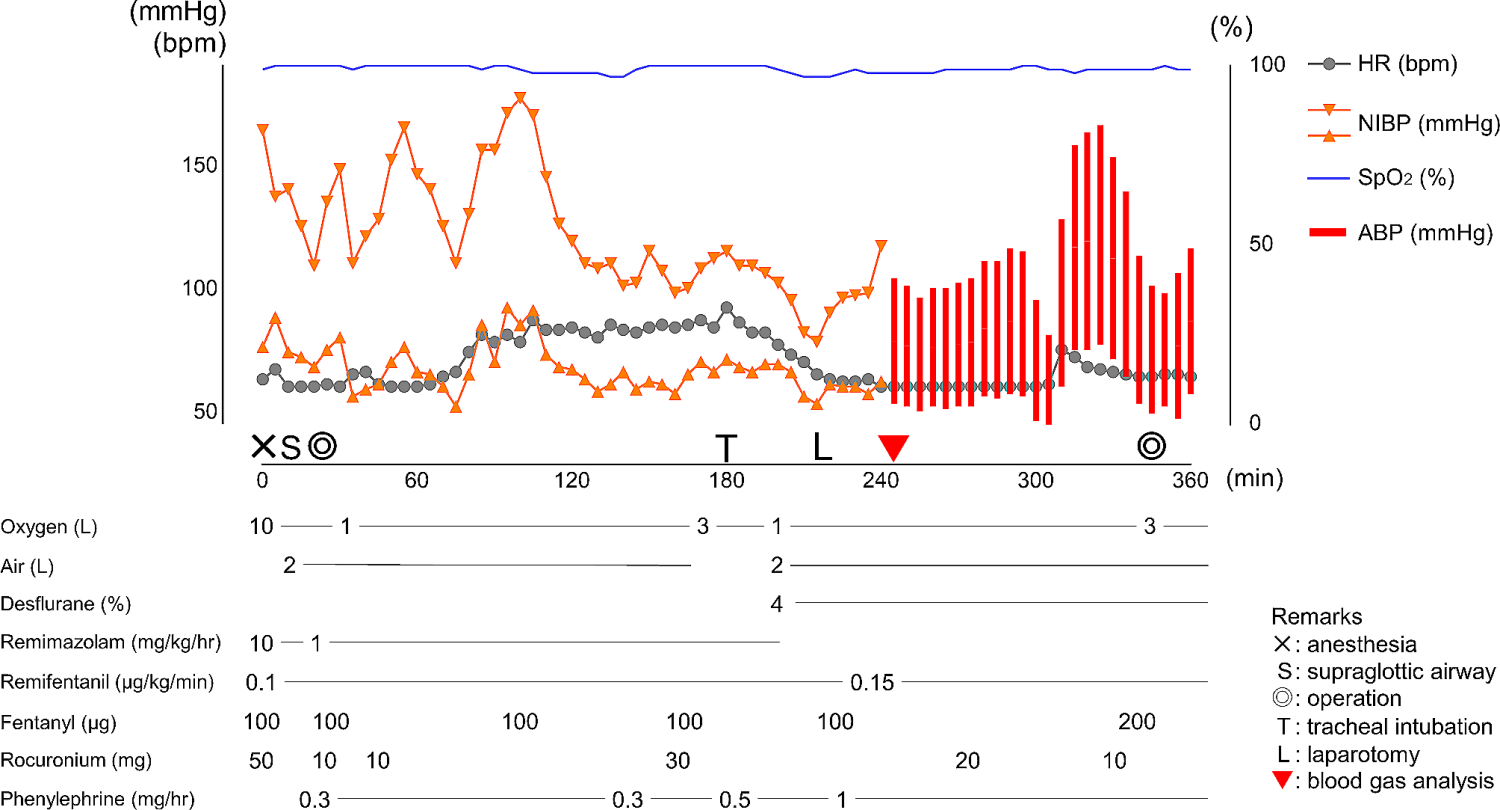
Anesthetic record of case 1 HR: Heart rate; NIBP: Non-invasive blood pressure; SpO_2_: Percutaneous oxygen saturation; ABP: Arterial blood pressure

A 76-year-old man (height, 166 cm; weight, 57 kg) was scheduled for transurethral resection of the prostate (TUR-P). He was taking enalapril maleate for hypertension and had a pacemaker implanted for an atrioventricular block due to myocarditis 4 years earlier. After general anesthesia was induced with 10 mg/kg/hr remimazolam, 0.1 µg/kg/min remifentanil, and 50 mg rocuronium, the patient’s airway was managed with a supraglottic airway device (SGA). Anesthesia was maintained with 1 mg/kg/hr remimazolam and 0.1 µg/kg/min remifentanil (Fig. 1). Saline was continuously irrigated during TUR-P in the lithotomy position. Irrigated saline was collected in a drape with a fluid collection pouch. Approximately 90 min after the surgery, the anesthesiologist noticed an air leak around the SGA and adjusted its position. After another 50 min, abdominal distension and cervical edema were observed. Venous blood gas analysis revealed a serum chloride concentration of 123 mEq/L. The anesthesiologist suspected saline IFA, and the patient’s trachea was intubated to secure the airway. After the intubation, anesthesia was maintained with 4% desflurane and 0.15 µg/kg/min remifentanil. Abdominal ultrasound examination indicated a subhepatic hypoechoic lesion, which suggested extravasation of saline. The surgeons suspected that the patient had a perforation of the bladder, and a laparotomy was subsequently performed to drain the leaked saline. Arterial blood gas analysis revealed hyperchloremia and acidemia (Table 1). The surgeons identified that the bladder neck was the site of perforation, which was where irrigated saline had leaked into the peritoneal space. Approximately 2 L of fluid were drained from the intraperitoneal and retroperitoneal spaces. The duration of the operation was 5 h and 26 min. The volume of saline used for irrigation was 28 L, and the estimated volume of deficit saline was approximately 5 L. The patient was sedated and transferred to the intensive care unit (ICU) under mechanical ventilation. He was extubated 2 h after the operation with no subsequent airway problems. The patient’s acidemia and electrolyte imbalance gradually resolved, and he was then transferred to the general ward on postoperative day 1. 

**Table 1:** Blood examination

	Preoperative	Intraoperative	Postoperative 1	Postoperative 2
Case 1	Venous blood	Arterial blood	Arterial blood	Arterial blood
FiO_2_		40	40	24
pH		7.191	7.266	7.361
PaCO_2_ (mmHg)		47.7	38.6	32.9
PaO_2_ (mmHg)		127	161	81.5
HCO_3_^−^ (mmol/l)		17.6	17.5	19.3
Hb (g/dl)	11.5	9.5	10.4	10.0
Na (mmol/l)	139	141	142	139
Cl (mmol/l)	106	124	118	112
Base excess		-9.8	-7.7	-5.0
anion gap		4.6	10.9	11.1
Case 2	Venous blood	Arterial blood	Arterial blood	Arterial blood
FiO_2_		30	30	30
pH		7.208	7.250	7.338
PaCO_2_ (mmHg)		46.1	43.6	40.3
PaO_2_ (mmHg)		111	138	184
HCO_3_^−^ (mmol/l)		17.7	18.4	21.1
Hb (g/dl)	12.6	9.1	8.7	8.2
Na (mmol/l)	142	144	143	141
Cl (mmol/l)	106	120	120	114
Base excess		-9.3	-7.8	-3.8
anion gap		10.2	8.7	9.8

**Fig. 2:**
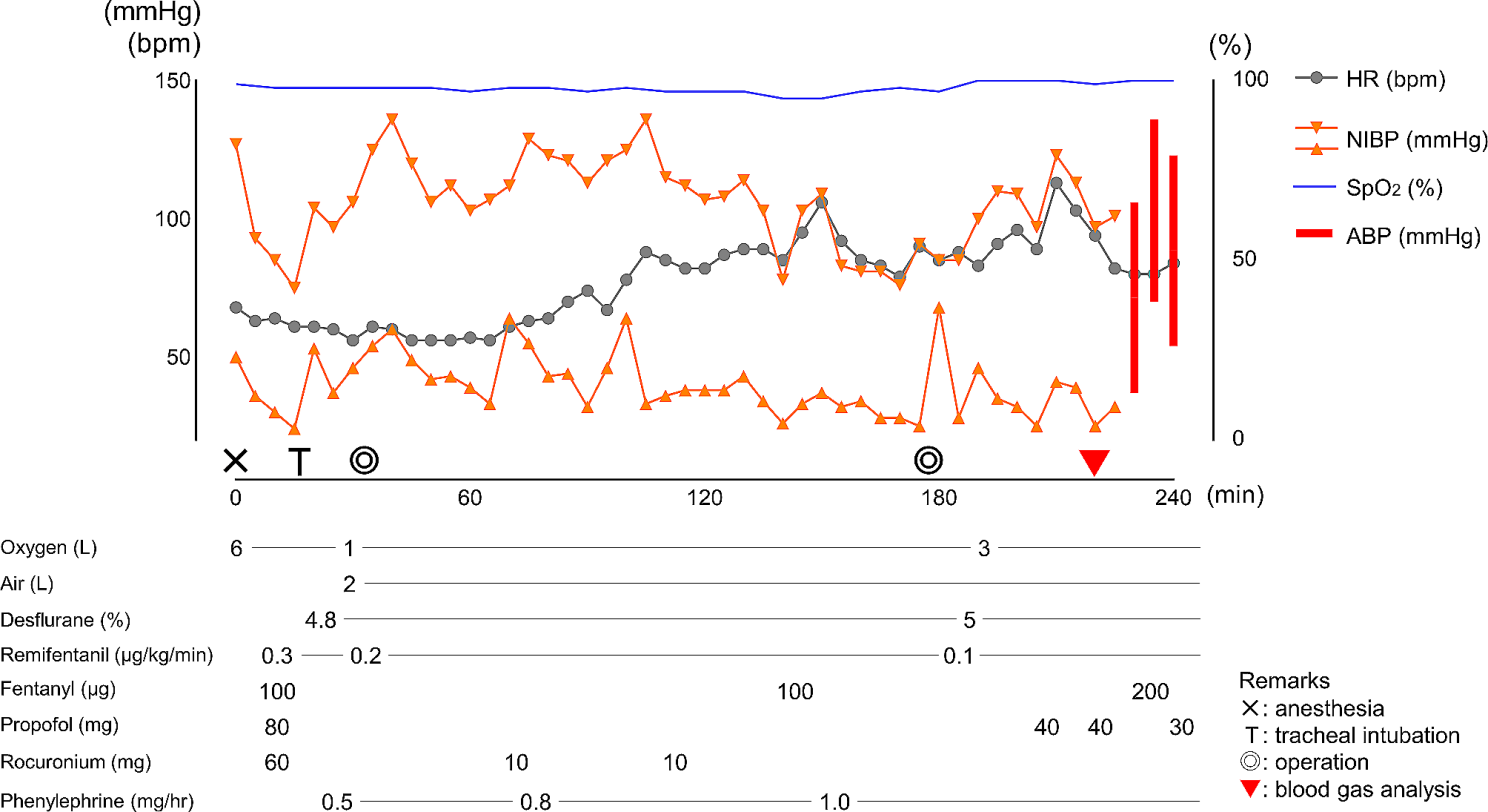
Anesthetic record of case 2 HR: Heart rate; NIBP: Non-invasive blood pressure; SpO_2_: Percutaneous oxygen saturation; ABP: Arterial blood pressure

### Patient 2

A 43-year-old woman (height, 165 cm; weight, 63 kg) was scheduled for transcervical resection (TCR) of a uterine fibroid. Preoperative magnetic resonance imaging indicated a 36 × 26 mm submucosal fibroid in the body of the uterus. She had no relevant medical history, and all her preoperative blood laboratory test results were normal. After general anesthesia was induced with 80 mg propofol, 0.3 µg/kg/min remifentanil, and 60 mg rocuronium, the trachea was intubated. Anesthesia was maintained with 4-5% desflurane and 0.2 µg/kg/min remifentanil. Transcervical resection of the uterine fibroid was performed with continuous saline irrigation of the uterus in the lithotomy position (Fig. 2). Irrigated saline was collected in a drape with a fluid collection pouch. After induction of anesthesia, continuous infusion and intermittent bolus injection of phenylephrine were required to manage mild hypotension. No other arrhythmia or hypoxia requiring therapeutic intervention occurred during the operation. The duration of the operation was 2 h and 20 min. At the end of the operation, the total amount of saline used for uterine irrigation was 26 L, and the estimated volume of deficit saline was approximately 4 L. When the drape was removed, notable upper extremity edema was observed, and venous blood gas analysis revealed a serum chloride concentration of 124 mEq/L. An arterial line was promptly inserted, and arterial blood gas analysis revealed hyperchloremia and acidemia (Table 1). In consideration of potential upper airway obstruction owing to neck subcutaneous edema, the patient was sedated and transferred to the ICU without being extubated. Furosemide was administered after admission to the ICU. The patient’s acidemia, electrolyte imbalance, and neck edema gradually resolved, and she was extubated 16 h after the operation without subsequent airway problems. She was then transferred to the general ward on postoperative day 1. 

## Discussion

In conventional TUR or TCR surgery with monopolar electrocautery, TUR syndrome caused by nonelectrolyte IFA has been a major concern for surgeons and anesthesiologists. In recent years, endoscopic systems that allow bipolar electrocautery have been developed, which therefore allow irrigation with normal saline. This new endoscopic system has eliminated the occurrence of TUR syndrome caused by nonelectrolyte IFA. However, there have been reports of other intraoperative complications caused by saline irrigation, namely, hyperchloremic metabolic acidosis [3, 4].

Intravascular or intraperitoneal absorption of a large volume of normal saline can lead to acidemia due to hyperchloremic metabolic acidosis, and disruption of the acid–base balance can cause a crisis in anesthetic management [5]. Acute metabolic acidosis is associated with increased morbidity and mortality rates as a result of significant hemodynamic deterioration, cardiac arrhythmias, increased inflammation, and an impaired immune response [6]. In addition to electrolyte and acid‒base imbalances, anesthesiologists should be aware of the potential development of head and neck edema and pulmonary edema as a result of volume overload. Like in the present case, saline IFA during HoLEP (holmium laser enucleation of the prostate) has been reported to cause notable head and neck edema, which requires subsequent tracheal intubation [7]. Additionally, pulmonary edema during TCR has been reported [8, 9]. 

There are 2 principal routes of absorption into the body: the vascular bed into blood vessels and through extravasation into the peritoneal or retroperitoneal space via the site of organ perforation [4]. According to the British Society for Gynecological Endoscopy and European Society for Gynecological Endoscopy guidelines, the deficit of irrigated fluid should be closely monitored and the maximum limit of saline absorption in healthy patients should be set at 2500 mL [10]. For elderly patients with cardiovascular, renal, or other comorbidities, the maximum saline absorption volume should be decreased and set to 1500 mL [10]. In the present study, 5 L of saline leaked into the peritoneum mainly via a perforation in the bladder (patient 1), whereas 4 L of saline was absorbed via the vascular bed of the uterus (patient 2), leading to significant acidemia. Risk factors for IFA syndrome include a prolonged surgery duration, large irrigation volume, height of the irrigation solution bag exceeding 60 cm, and high intravesical or intrauterine pressure [5]. 

In conventional endoscopic surgery, nonelectrolyte fluid is used, and spinal anesthesia is the primary choice for the early detection of TUR syndrome symptoms. However, saline irrigation during endoscopic surgery does not increase the incidence of TUR syndrome development so general anesthesia could be a feasible alternative. Okuma et al. reported a case of saline IFA with persistent hypotension, disturbance of consciousness, and severe acidemia in a patient undergoing TUR-P under spinal anesthesia [11]. Given the goal of early symptom detection, spinal anesthesia remains a rational choice, even in endoscopic surgeries with saline irrigation. In addition to the patient’s vital signs and level of consciousness, intraoperative monitoring of the in-out balance of irrigation fluid and intravesical or intrauterine pressure during the surgical procedure are effective practices for preventing IFA symptoms [7, 10]. 

In the present study, an arterial line was inserted immediately after identifying saline IFA, allowing us to evaluate the electrolyte and acid‒base balance as well as the oxygenation status. In Patient 1, the presence of an echo-free space on abdominal ultrasonography was useful for detecting saline leakage into the peritoneal cavity. In Patient 2, head and neck edema caused by volume overload posed a risk of upper airway obstruction, which indicated the need for tracheal intubation. In patients with a large volume of saline IFA and severe acidemia, it is advisable to transfer the patient to the ICU or high care unit (HCU) postoperatively. Rigorous management of fluid balance, respiration, and blood pressure must continue into the postoperative period to allow the progressive normalization of any electrolyte or acid–base imbalances. The administration of diuretics, such as a loop diuretic, is a viable treatment option for managing electrolyte imbalance and volume overload [10].

Intraoperative IFA is rare, and its incidence rate in transurethral or transcervical endoscopic surgeries is unclear. In our institution, TUR or TCR surgeries that use bipolar electrocautery and normal saline irrigation have been performed since 2009. The two cases we currently present are the first instances of IFA in our experience. Given the complexities observed in these cases, it’s important to recognize that the transurethral and transcervical surgeries undertaken were far from routine, marked by their prolonged and intricate nature. Each case presented substantial challenges, such as unforeseen operative complications and unusually long surgical times, diverging significantly from standard endoscopic procedures. 

In conclusion, we report 2 cases in which substantial absorption of irrigated saline led to hyperchloremic metabolic acidosis. The widespread use of endoscopic systems that use bipolar electrocautery and normal saline irrigation in transurethral or transcervical endoscopic surgery has effectively decreased the incidence of complications such as TUR syndrome. However, anesthesiologists should remain vigilant in detecting metabolic acidemia, cardiopulmonary complications, and airway obstruction caused by excessive saline absorption. Arterial blood gas analysis is useful in diagnosing saline IFA, while abdominal ultrasonography can be helpful in identifying intraperitoneal leakage of irrigation fluid. Postoperatively, careful monitoring and care, including diuresis, electrolyte correction, and respiratory management, are necessary. 

## Data Availability

The anesthesia records and laboratory data used in the present study are available from the corresponding author upon reasonable request.

## Abbreviations

IFA: irrigation fluid absorption
TUR: transurethral resection
TUR-P: transurethral resection of the prostate
SGA: supraglottic airway
ICU: intensive care unit
TCR: transcervical resection
HoLEP: holmium laser enucleation of the prostate
HCU: high care unit
